# Cutaneous Leishmaniasis of Lip and Role of Polymerase Chain Reaction: A Case Report

**DOI:** 10.31729/jnma.5025

**Published:** 2020-07-31

**Authors:** Niraj Parajuli, Krishna Das Manandhar, Srijan Shrestha, Anup Bastola

**Affiliations:** 1 Department of Dermatology and Venereology, National Academy of Medical Sciences, Bir Hospital, Kathmandu, Nepal; 2 Central Department of Biotechnology, Tribhuvan University, Kathmandu, Nepal; 3 Department of Tropical Medicine, Dermatology and Sexually Transmitted Infections, Sukraraj Tropical and Infectious Disease Hospital, Teku, Kathmandu

**Keywords:** *cutaneous leishmaniasis*, *lip*, *mucocutaneous*, *polymerase chain reaction*

## Abstract

The diagnosis of cutaneous leishmaniasis is mostly confirmed by the identification of parasite in a skin smear or biopsy. However, this method may not always be sensitive enough to detect the disease when parasitic load is low. Molecular test such as polymerase chain reactions can be useful in such circumstances. Here, we report a case of cutaneous leishmaniasis diagnosed by a polymerase chain reaction test when both smear and biopsy failed to confirm the diagnosis. A 17-years-old female from mountainous district of Nepal, presented with a crusted plaque over the upper lip for a duration of 6 months. Both skin smear and biopsy from the lesion failed to demonstrate Leishmania parasite but a polymerase chain reaction test was positive for Leishmania donovani. This case emphasizes on the importance of molecular testing such as polymerase chain reaction when commonly performed diagnostics test fails to support confirmation of clinical diagnosis.

## INTRODUCTION

Leishmaniasis are a group of parasitic diseases caused by Leishmania species and transmitted through the bite of an infected female sandfly. The disease manifest itself in three clinical forms, namely cutaneous, mucocutaneous, and potentially fatal visceral forms.^1^ The diagnosis is confirmed by the identification of the parasite in smear, biopsy, or tissue culture.^2^ Parasitological diagnosis is considered as the gold standard because of its high specificity.^3^ However, molecular test such as polymerase chain reaction (PCR) which is not readily available in our setting is useful for the detection of low amounts of deoxyribonucleic acid (DNA) in tissues.^4,5^

## CASE REPORT

A 17-years old unmarried female from Bajura district of Nepal came to the dermatology out-patient department with a complaint of a single plaque over the right upper lip for the last 6 months. Lesion started as a small red papule which gradually increased in size with central crusting. No history of fever or weight loss was given. There was no significant family or medical history. Multiple over the counter treatment was used as trial without relief. There was no history of travel outside of her village until the time for consultation.

**Figure 1.**
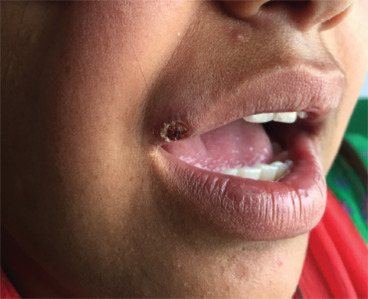
An ill-defined crusted plaque over upper lip.

On examination, a single round to oval crusted plaque approximately 1.5 × 1.5 cm was noted over the right upper lip. Neither an induration nor easy bleeding was noted. No regional lymphadenopathy was present (Figure 1).

A slit skin examination was done for acid-fast and Giemsa stain which was negative for any organism. An incisional biopsy was done which showed multiple epitheloid type multinucleated giant cells suggesting Lupus vulgaris. However, a mantoux test revealed an induration of only 2 mm in 48 hours.

**Figure 2.**
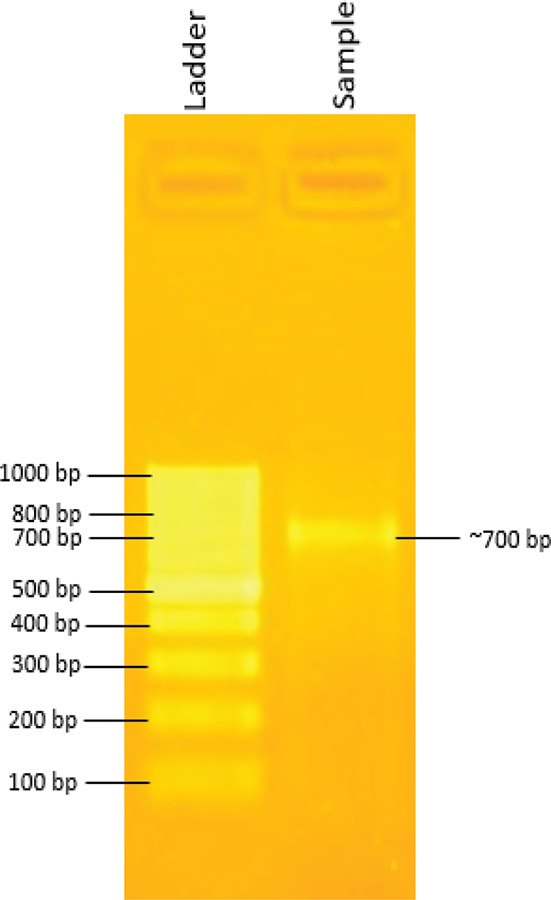
Figure showing 1.5% agarose gel electrophoresis for PCR amplicon, ladder - 100 bp ladder (Gene ruler, Cat. No. SM0243). Sample-PCR amplicon obtained after 2^nd^ round of PCR.

The morphology of lesion prompted us to investigate furthermore for cutaneous leishmaniasis before starting on anti-tubercular medications. We decided to do PCR for Leishmania species from the lesion. The sample was sent to the Laboratory of the Central Department of Biotechnology, Tribhuvan University, Kathmandu, Nepal due to unavailability of PCR in the National Academy of Medical Sciences, Bir hospital. PCR was done using the nested protocol previously described by Noyes, et al. in 1998.^6^ The DNA template for PCR was extracted manually from the lesional tissue. After second-round PCR, a band size corresponding to the size of approximately 700 bp was obtained that confirmed the presence of Leishmania parasite (Figure 2). The band size corresponded to that typical of Leishmania donovani.

A serological test conducted with an rk39 rapid diagnostic test kit also tested positive for this case.

An ultrasound of the abdomen and pelvis was normal. Liver function tests, renal function test, and blood sugar were also all within normal limits.

**Figure 3.**
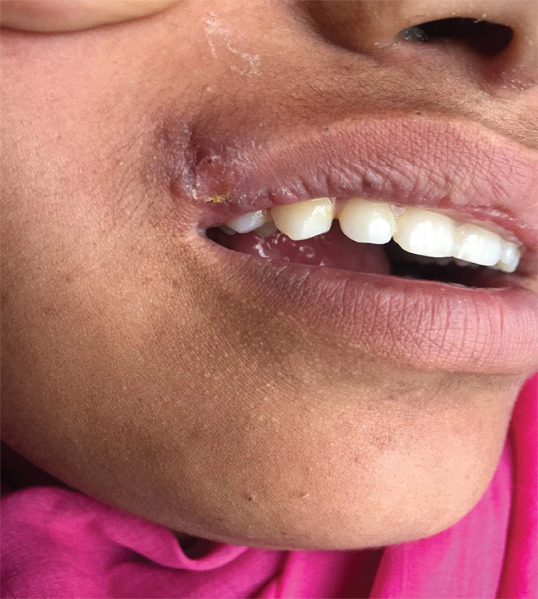
Follow-up in one month with spontaneous healing of plaque over upper lip.

Patient was diagnosed as a case of cutaneous leishmaniasis and was advised for follow-ups without any treatment. The patient came for follow-up in one month's time where the plaque had decreased significantly (Figure 3). The patient was advised for regular follow-ups on a monthly basis till the lesion subsided. The lesion healed spontaneously within a couple of months.

## DISCUSSION

Cutaneous leishmaniasis (CL) is the most common type of leishmaniasis. Nepal is a non-endemic region for CL even though it is endemic for visceral and post kala-azar dermal leishmaniasis (PKDL).^7^ CL typically presents as crusted lesion mostly over the exposed part of the body which generally heals within 3-6 months duration.^8^ The case presented above is from a non-endemic district for visceral leishmaniasis. It is important to make an accurate diagnosis of CL to prevent unnecessary treatment and scar formation.^9^ The diagnosis is made by visualization of the amastigote form of Leishmania microscopically. PCR has shown to be more sensitive in detecting Leishmania species when the DNA load is small.^5^

Most CL patients can be managed with topical treatments only. On the contrary, some Leishmania species can cause mucocutaneous involvement requiring a systemic therapeutic approach. Moreover, Leishmania species can vary in their sensitivity to available therapeutic modalities. This makes species determination critical for the choice of treatment and the clinical outcome of CL. Identification of the Leishmania species used to be laborious, but now it can be identified easily with new DNA techniques that will enable a more rational therapy choice.^3^

The majority of the cases are being diagnosed by either a slit skin smear or fine-needle aspiration with Giemsa stain. This case emphasizes the importance of more sensitive tests like PCR in diagnosing CL when there is a strong clinical suspicion.
